# WIF1 prevents Wnt5A mediated LIMK/CFL phosphorylation and adherens junction disruption in human vascular endothelial cells

**DOI:** 10.1186/s12950-017-0157-4

**Published:** 2017-05-19

**Authors:** Tom Skaria, Esther Bachli, Gabriele Schoedon

**Affiliations:** 1 Inflammation Research Unit, Division of Internal Medicine, University Hospital Zürich, Rämistrasse 100, CH-8091 Zürich, Switzerland; 2 Department of Medicine, Uster Hospital, Brunnenstrasse 42, CH-8610 Uster, Switzerland

**Keywords:** Inflammation, Endothelial permeability, Wnt5A, WIF1, Ryk, Cytoskeleton remodeling

## Abstract

**Background:**

Wnt5A is released by activated macrophages and elevated levels have been detected in sepsis patients with severe systemic inflammation. However, the signalling and functional effects of Wnt5A in the vascular endothelial cells (VEC) remained unclear. Recently, we showed that Wnt5A affects barrier function in human VEC through Ryk interaction. Wnt5A/Ryk signalling activates LIMK to inactivate the actin depolymerisation factor CFL by phosphorylation, promotes actin polymerisation and disrupts endothelial adherens junctions.

**Findings:**

Here, we investigate the antagonistic effect of the Ryk specific secreted Wnt antagonist Wnt inhibitory factor (WIF)-1 on Wnt5A-mediated activation/inactivation of LIMK/CFL, and adherens junction disruption in human VEC. In human coronary artery endothelial cells (HCAEC), treatment with Wnt5A enhanced the phosphorylation of LIMK and CFL that was significantly prevented by WIF1. The presence of WIF1 suppressed Wnt5A-mediated disruption of β-catenin and VE-cadherin adherens junctions in HCAEC, thereby preventing barrier dysfunction caused by Wnt5A.

**Conclusion:**

We conclude that WIF1 or molecules with similar properties could be potent tools for the prevention of vascular leakage due to Wnt5A-mediated actin cytoskeleton remodeling in diseases associated with systemic inflammation.

**Electronic supplementary material:**

The online version of this article (doi:10.1186/s12950-017-0157-4) contains supplementary material, which is available to authorized users.

## Background

Vascular endothelial cells (VEC) in physiologic state play a crucial role in maintaining vascular homeostasis. They preserve blood fluidity [1, 2], control blood pressure, prevent the adherence of leukocytes [1] and platelets [2], and regulate vascular permeability [1]. In diseases associated with inflammatory states, vascular leakage is a major pathophysiologic event. In systemic inflammation and sepsis, as well as in autoimmune and allergic inflammation, activated blood components and inflammatory mediators such as cytokines act upon VEC and heavily alter their functions [2], conferring an inflamed phenotype. Inflamed VEC are procoagulant and exhibit increased production of vasoactive substances, expression of cell adhesion molecules, synthesis of inflammatory mediators including chemoattractants, and barrier dysfunction causing leakage [1].

Wnt5A, a lipid modified signalling protein of the non-canonical Wnt family [3] is detected in high levels in sera and bone marrow of patients with severe systemic inflammation and septic shock [4, 5]. Its expression has also been found elevated in macrophage-accumulated intima from human and murine atherosclerotic lesions [6]. Wnt5A is a chemokine secreted by Toll-like receptor-activated human macrophages, and is crucially involved in sustaining their inflammatory response [4, 7]. However, the targets and functional effects of Wnt5A on human VEC remained unclear.

Recently, we identified a novel Wnt5A signalling mechanism affecting monolayer integrity and barrier function [8] in our established in vitro system of primary immunocompetent VEC, human coronary artery endothelial cells (HCAEC) [9]. Wnt5A targets the Rho-associated protein serine/threonine kinase (ROCK) to phosphorylate LIMK2 and actin depolymerisation factor CFL1, thereby increasing actin stress fiber formation. This disrupts β-catenin and VE-cadherin at adherens junctions (AJ) leading to inter-endothelial gap formation and enhanced endothelial monolayer permeability. To mediate its effects on endothelial barrier function, Wnt5A has been found to act through Ryk receptor as silencing Ryk expression prevented Wnt5A-induced hyperpermeability [8]. Antagonising Wnt5A activity with Ryk specific Wnt inhibitory factor (WIF) -1 [10] prevents Wnt5A-induced actin stress fiber formation [8, 11] while Fzd or Ror specific secreted Frizzled-related peptide (sFRP) [10] is ineffective [8, 11].

However, the ability of the natural Wnt antagonists sFRP1 and WIF1 to antagonize LIMK/CFL phosphorylation and AJ disruption, the cellular events occurring upstream and downstream of enhanced actin stress fiber formation upon Wnt5A treatment in HCAEC need to be still verified. Here, we investigated the effects of sFRP1 and WIF1 on the phosphorylation of LIMK and CFL in human coronary artery endothelial cells (HCAEC) in the presence of Wnt5A.

## Methods

### Cell culture

HCAEC (Cat. No. CC-2585, Clonetics, Lonza, Switzerland) were propagated in EBM-2 medium (Clonetics, Lonza) supplemented with EGM-2MV Single Quots with 5% FBS (Clonetics, Lonza) [9]. HCAEC used in this study were tested positively and functionally for CD31/105, von Willebrand Factor VIII, and acetylated low density lipoprotein uptake as certified by the manufacturer (Lonza). Additionally, we confirmed stable CD31 expression by HCAEC under experimental conditions (Additional file 1: Figure S1). Cells were treated with recombinant human/mouse Wnt5A (250 ng/mL, R&D systems), recombinant human WIF1 (15 μg/mL, R&D systems) or recombinant human sFRP1 (10 μg/mL, R&D systems) [8]. Experiments were performed using cells from passages 3–6.

### Immunoblotting

Immunoblotting of pLIMK, LIMK, pCFL and CFL and quantification of band densities were performed as described [11]. The following primary antibodies were used with dilutions indicated: rabbit anti-phospho LIMK2, polyclonal (1:500, Abcam), rabbit anti-LIMK2, polyclonal (1:1000, ThermoFischer Scientific), rabbit anti-phospho CFL, polyclonal (1:1000, Abcam), rabbit anti- CFL, polyclonal (1:1000, Abcam). Anti-rabbit IgG- HRP-linked whole antibody (GE Healthcare UK Limited) was used as the secondary antibody, diluted 1:5000.

### Immunofluorescence staining

Immunofluorescence staining for β-catenin and VE-cadherin were performed as described [8]. The following primary antibodies were used with dilutions indicated: rabbit-anti- β-catenin (1:150, Cell Signalling Technology), rabbit-anti-VE-cadherin (1:100, Cell Signalling Technology), rabbit-anti-CD31 (1:40, Epitomics). Alexa 568 labelled goat anti-rabbit antibody was used as the secondary antibody, diluted 1:2000. The cellular fluorescence intensities of β-catenin and VE-cadherin at inter-cellular boarders were quantified from five different fields (covering approx. 90% of total cells/field) using ImageJ based Fiji software (Fiji is Just ImageJ) and corrected for background fluorescence.

### Statistical analysis

Data were analysed using GraphPad Prism software version 5.04 (GraphPad Software, San Diego, CA). An unpaired 2-tailed Student’s *t*-test or for comparing data among groups, 1-way ANOVA followed by the Newman-Keuls test was used and differences were considered statistically significant at *P* < 0.05.

## Results and discussion

**Fig. 1:**
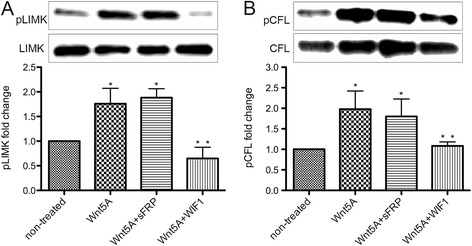
Effects of the Wnt antagonists WIF1 and sFRP1 on Wnt5A-mediated phosphorylation of LIMK and CFL in VEC. Immunoblot of (**a**) pLIMK, LIMK and (**b**) pCFL and CFL in HCAEC treated with Wnt5A alone and in combination with either WIF1 or sFRP1 (sFRP) for 1 and 4 h, respectively. Bands from left to right, non-treated cells; Wnt5A-treated cells; Wnt5A + sFRP1 treated cells; Wnt5A + WIF1 treated cells. The ratios of (**a**) pLIMK2/LIMK2, and (**b**) pCFL/CFL were calculated from densitometry analyses. Data are mean ± SEM from three independent experiments. *P < 0.05 vs non-treated, **P < 0.05 vs Wnt5A

**Fig. 2:**
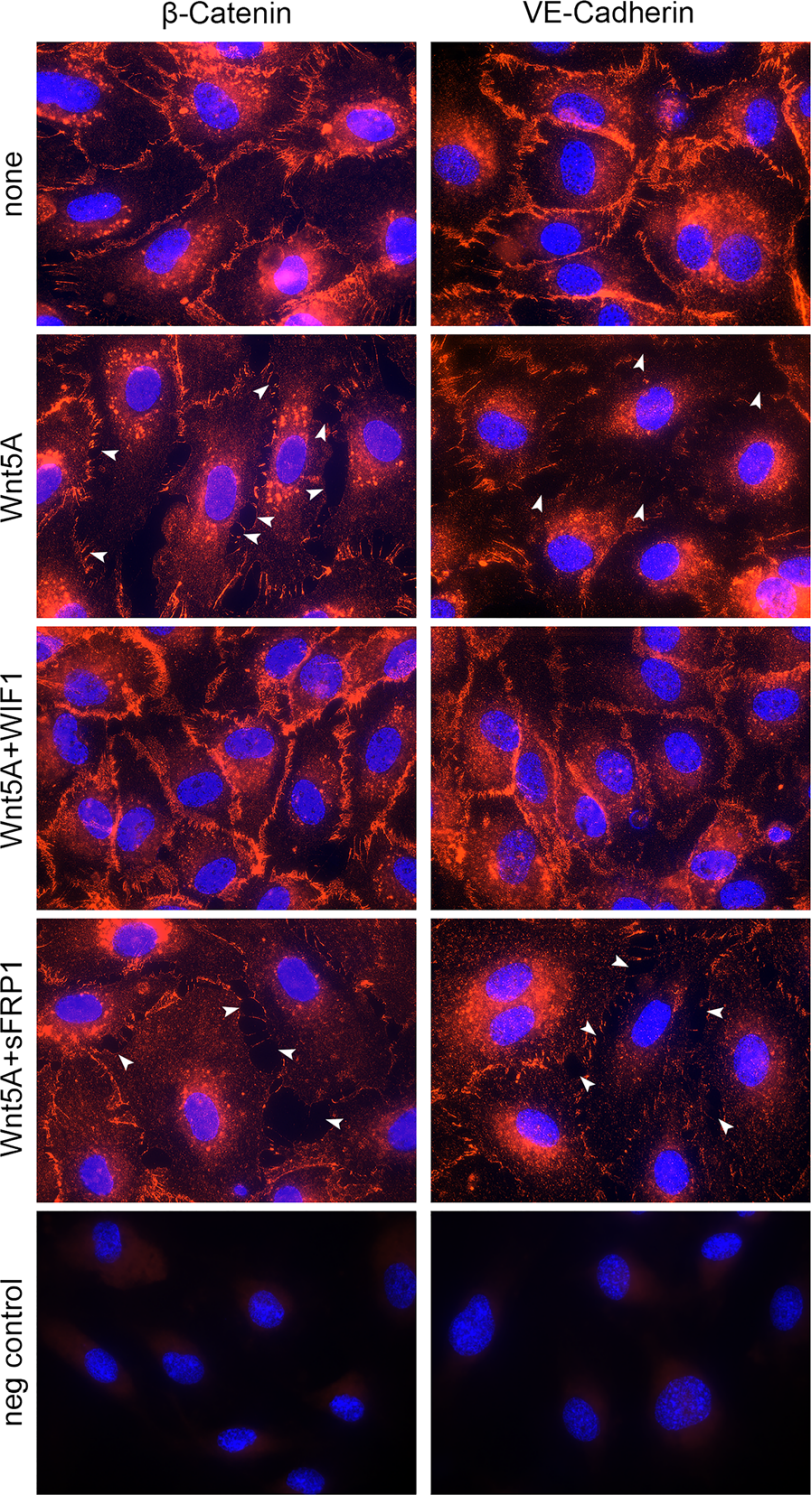
AJ assembly in VEC treated with Wnt5A in the presence or absence of WIF1 and sFRP1. Immunofluorescence staining for β-catenin and VE-cadherin (*red*) in HCAEC treated with Wnt5A either alone or in the presence of WIF1 and sFRP1 for 8 h. *Blue stain* (DAPI) indicates nuclei. Arrow heads point to inter-endothelial gaps. Zeiss Axioskope, original magnification 630 ×

Phosphorylation of LIMK/CFL were significantly increased in Wnt5A-treated HCAEC compared with non-treated cells (Fig. 1), thereby confirming previous findings [8]. Here, we further tested the antagonizing effects of WIF1 on Wnt5A-induced phosphorylation of LIMK/CFL. As shown herein by western blot analysis, combining Wnt5A with WIF1 significantly suppressed Wnt5A-triggered phosphorylation of both LIMK and CFL while combination with sFRP was not effective (Fig. 1). When activated by phosphorylation, LIMK phosphorylates the actin depolymerisation factor CFL1. Phosphorylation inactivates CFL1 and prevents its ability to depolymerise actin filaments leading to the formation of actin stress fibers. Increased stress fiber formation disrupts the AJ assembly at inter-endothelial junctions [12]. Here, we further investigated the antagonizing effects of WIF1 on Wnt5A-induced disruption of AJ. In non-treated cells, β-catenin and VE-cadherin were aligned at the cellular periphery forming intercellular boarders. Consistent with our previous finding [8], treatment with Wnt5A notably disrupted both β-catenin and VE-cadherin at intercellular boarders, forming large inter-endothelial gaps (Fig. 2, white arrow heads). Additionally, this study has shown that combining Wnt5A with WIF1 notably prevented Wnt5A-induced disassembly of both β-catenin and VE-cadherin while sFRP failed to exert any antagonistic effects (Fig. 2, Additional file 2: Figure S2A, B). This finding is supported by functional measurements of HCAEC monolayer permeability. Treatment with Wnt5A significantly decreased the resistance of HCAEC monolayers to alternating current, thereby indicating enhanced permeability of Wnt5A-treated HCAEC monolayers (Additional file 3: Figure S3), confirming previous findings [8]. Combining Wnt5A with WIF1 significantly prevented Wnt5A-induced endothelial hyperpermeability while no significant antagonistic effect was observed with sFRP1 (Additional file 3: Figure S3).

**Fig. 3:**
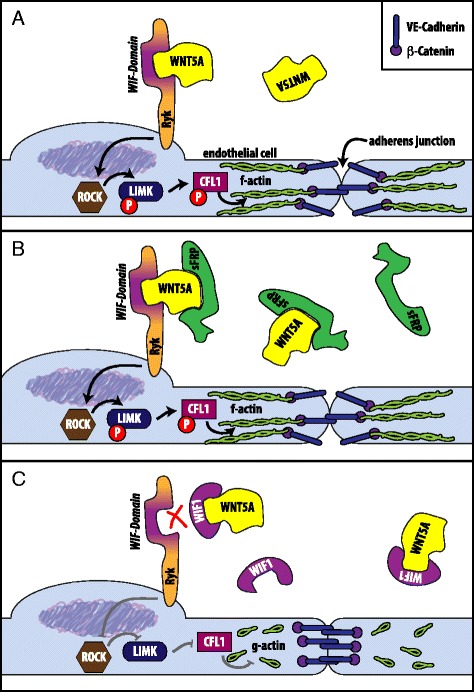
Interaction of antagonists with Wnt5A/Ryk signalling in vascular endothelial cells. **a** Wnt5A binds to the Ryk receptor by interaction with its WIF domain, inducing downstream activation of the ROCK/LIMK/CFL pathway. Phosphorylated CFL is inactivated and allows formation of actin stress fibers (f-actin), that can interact with adherens junction protein β-catenin and tears VE-cadherin junctions apart. As a result, endothelial monolayer permeability is increased. **b** In the presence of the Wnt antagonist secreted Frizzled-related peptide (sFRP) that covers the Wnt5A binding site for the cysteine-rich domain of Frizzled receptors, interaction of Wnt5A with Ryk is still unaffected. **c** In the presence of the Wnt antagonist WIF1 (WIF) that covers the Wnt5A binding site interacting with Ryk receptor’s WIF domain, Wnt5A/Ryk interaction is blocked, and downstream signalling is not transduced. CFL1 remains active and restricts f-actin formation. Actin remains mainly in the globular form (g-actin), that does not interact with adherens junction proteins

Ryk was previously identified as the Wnt5A receptor on HCAEC [8]. The current study demonstrates the potency of WIF1 to prevent Wnt5A-induced cytoskeleton remodelling and monolayer barrier dysfunction in human vascular endothelial cells. WIF1 is a 371 amino acid protein composed of a distinct WIF domain homologous to the Wnt binding WIF domain of the Ryk receptor, five EGF-like domains, and a hydrophilic tail [13]. Secreted Wnt antagonists including WIF1 and sFRP sequester Wnt proteins away from the receptors, thereby inhibiting signalling pathways [10, 13] (see Scheme, Fig. 3). It has been shown that the antagonistic effect of WIF1 is mainly mediated by its WIF domain. In an attempt to map the Wnt binding sites in WIF domain, previous studies revealed the presence of an alkyl-binding site that is capable of interacting with essential lipid groups of Wnts [14–16]. Although WIF1 is expressed in different tissues, higher levels are reported in cartilage, lung, retina and brain [13, 17–20]. Several studies link elevated expression level of Wnt5A to inflammatory response in sepsis and atherosclerosis [4–6, 21]. Inflammatory activated leucocytes are the major source of Wnt5A [4, 7] and in this context, the observation that elevated Wnt5A levels correlate positively with the leucocyte count in sepsis is of particular interest [5]. The presence of lower levels of sFRP observed during worsening of disease in sepsis patients [5] is of further interest as various sFRP are shown to attenuate Fzd receptor-mediated inflammatory Wnt5A signalling in leucocytes [4, 7]. A previous study showed the upregulation of WIF1 mRNA in pro-inflammatory activated human monocytes [4]. Presently, it is not clear if monocytes release WIF1 to counter regulate the adverse effects of Wnt5A on VEC. In light of recent in vitro findings supporting an inhibitory effect of WIF1 on Wnt5A signalling in human VEC, the altered expression and antagonistic effects of WIF1 in diseases showing dysregulated Wnt5A signalling is worthy of future investigations.

In conclusion, this is the first report identifying antagonistic effects of WIF1 on Wnt5A mediated actin cytoskeleton signalling pathway in primary human vascular endothelial cells. Our data suggest that the Wnt5A pathway leading to barrier dysfunction of vascular endothelial cells is a target for the natural Wnt5A antagonist WIF1. This finding could offer novel therapeutic options for diseases associated with severe vascular leakage such as sepsis and septic shock.

## Additional files

Additional file 1: Figure S1. Expression of CD31 in HCAEC. Immunofluorescence staining for CD31 protein (*red*) in HCAEC either untreated (none) or treated with Wnt5A or TNF-alpha (20 U/mL) for 8 h. Nuclei are stained blue (DAPI). Zeiss Axioskope, original magnification 630 × . (PDF 8535 kb)

Additional file 2: Figure S2. β-catenin and VE-cadherin expression at inter-cellular boarders. Mean fluorescence intensities of (**a**) β-catenin and (**b**) VE-cadherin at inter-cellular boarders quantified using ImageJ based Fiji software. Data are mean ± SEM from three independent experiments. *P < 0.05 vs non-treated, **P < 0.05 vs Wnt5A. (ZIP 1032 kb)

Additional file 3: Figure S3. Barrier function of Wnt5A-treated VEC in the presence or absence of WIF1 and sFRP1. **a** ECIS assisted measurements (Additional file 4: supplementary methods) showing resistance of HCAEC monolayers grown in 8W10E+ arrays treated with vehicle (*black*), Wnt5A (*green*), Wnt5A + WIF1 (*yellow*) and Wnt5A + sFRP1 (*purple*). Data shown are the resistance measurements conducted at 4000 Hz and are mean ± SEM of 2 wells from 1 out of three representative experiments. **b** Barrier function measurements indicating the significance of WIF1’s antagonistic effect on Wnt5A and are mean ± SEM of three independent experiments run with duplicate wells. *P < 0.05 vs non-treated, **P < 0.05 vs Wnt5A. (PDF 2125 kb)

Additional file 4: Supplementary methods. (PDF 300 kb)

## Abbreviations

AJ: Adherens junctions
HCAEC: Human coronary artery endothelial cells
sFRP: Secreted frizzled-related peptide
VEC: Vascular endothelial cells
WIF1: Wnt inhibitory factor −1
