# Supplementary figures and images for: WIF1 prevents Wnt5A mediated LIMK/CFL phosphorylation and adherens junction disruption in human vascular endothelial cells
Source: J Inflamm (Lond). 2017 May 19;14:10. doi: 10.1186/s12950-017-0157-4 (PMC5437570; doi:10.1186/s12950-017-0157-4)

**none**

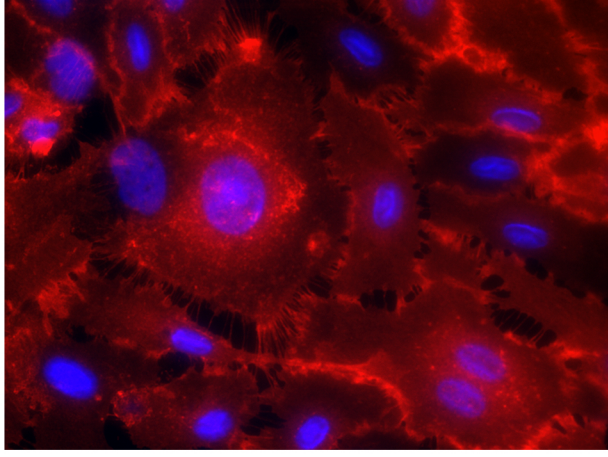

**Wnt5A**

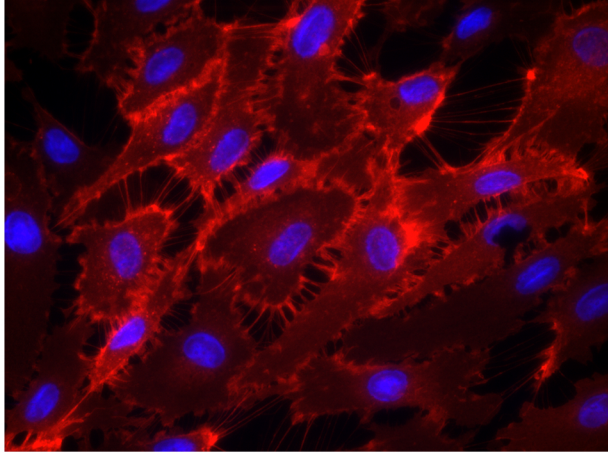

**TNF-alpha**

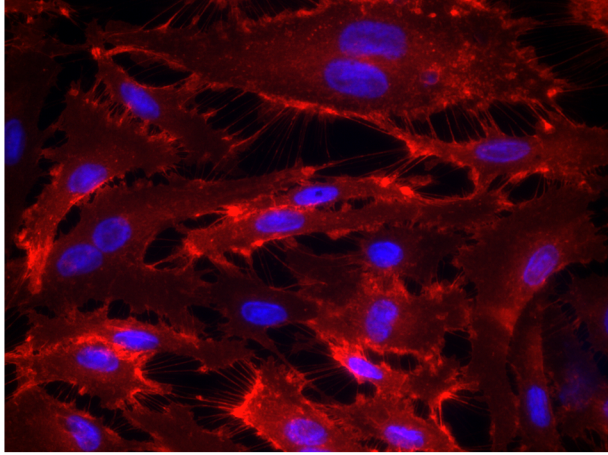

Supplement: Supplementary file 1 — Expression of CD31 in HCAEC. Immunofluorescence staining for CD31 protein (red) in HCAEC either untreated (none) or treated with Wnt5A or TNF-alpha (20 U/mL) for 8 h. Nuclei are stained blue (DAPI). Zeiss Axioskope, original magnification 630 × . (PDF 8535 kb) [file 12950_2017_157_MOESM1_ESM.pdf]

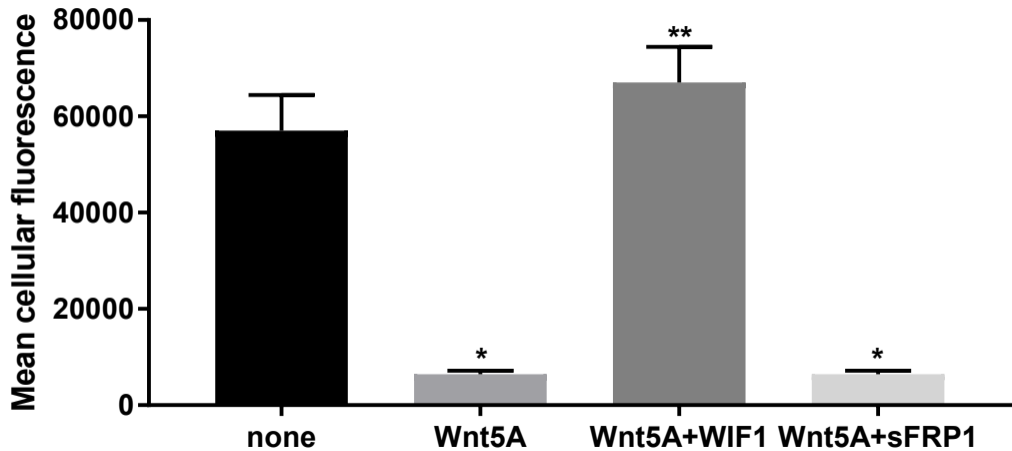

Supplement: Supplementary file 2 — β-catenin and VE-cadherin expression at inter-cellular boarders. Mean fluorescence intensities of (a) β-catenin and (b) VE-cadherin at inter-cellular boarders quantified using ImageJ based Fiji software. Data are mean ± SEM from three independent experiments. *P < 0.05 vs non-treated, **P < 0.05 vs Wnt5A. (ZIP 1032 kb) [file 12950_2017_157_MOESM2_ESM.zip › Figure S2A.pdf]

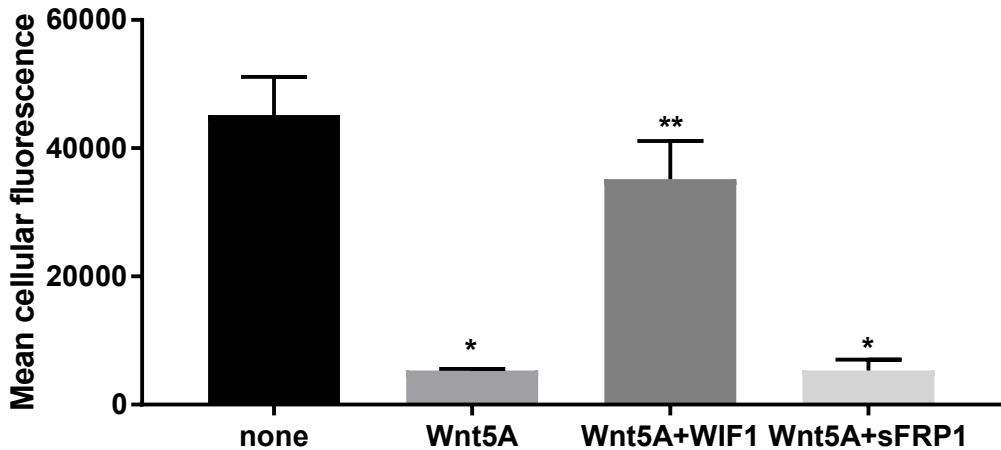

Supplement: Supplementary file 2 — β-catenin and VE-cadherin expression at inter-cellular boarders. Mean fluorescence intensities of (a) β-catenin and (b) VE-cadherin at inter-cellular boarders quantified using ImageJ based Fiji software. Data are mean ± SEM from three independent experiments. *P < 0.05 vs non-treated, **P < 0.05 vs Wnt5A. (ZIP 1032 kb) [file 12950_2017_157_MOESM2_ESM.zip › Figure S2B.pdf]

**A**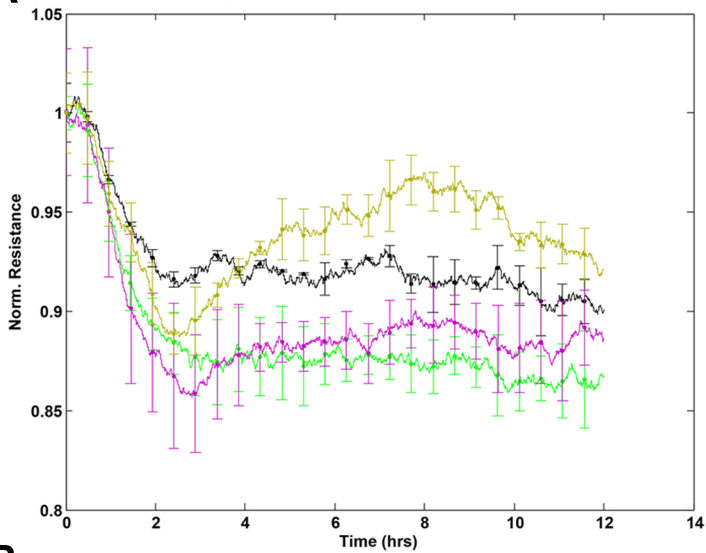**B**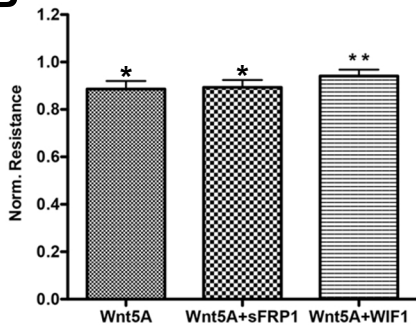

Supplement: Supplementary file 3 — Barrier function of Wnt5A-treated VEC in the presence or absence of WIF1 and sFRP1. a ECIS assisted measurements (Additional file 4: supplementary methods) showing resistance of HCAEC monolayers grown in 8W10E+ arrays treated with vehicle (black), Wnt5A (green), Wnt5A + WIF1 (yellow) and Wnt5A + sFRP1 (purple). Data shown are the resistance measurements conducted at 4000 Hz and are mean ± SEM of 2 wells from 1 out of three representative experiments. b Barrier function measurements indicating the significance of WIF1’s antagonistic effect on Wnt5A and are mean ± SEM of three independent experiments run with duplicate wells. *P < 0.05 vs non-treated, **P < 0.05 vs Wnt5A. (PDF 2125 kb) [file 12950_2017_157_MOESM3_ESM.pdf]
