# Supplementary material for: WIF1 prevents Wnt5A mediated LIMK/CFL phosphorylation and adherens junction disruption in human vascular endothelial cells
Source: J Inflamm (Lond). 2017 May 19;14:10. doi: 10.1186/s12950-017-0157-4 (PMC5437570; doi:10.1186/s12950-017-0157-4)
Supplement: Supplementary file 4 — Supplementary methods. (PDF 300 kb) [file 12950_2017_157_MOESM4_ESM.pdf]

<u>Additional file 4</u>

<u>Supplementary Methods</u>

Endothelial barrier function analysis

Endothelial barrier function in response to a specific stimulus can be measured in a fully standardized manner by continuously recording changes in trans-endothelial electric resistance using Electric Cell-substrate Impedance Sensing (ECIS) [1, 2]. The barrier function of HCAEC was recorded in real-time using the ECIS Z Theta system (Applied Biophysics) and the associated software v.1.2.126 PC, as described [3]. HCAEC seeded (80,000- 90,000 cells/well) into rat tail collagen type I coated and stabilized 8W10E+ electrode chamber arrays were grown up to tight monolayer stage. Treatments were then carried out by replacing the medium with fresh medium without or with stimuli (Wnt5A, Wnt5A+sFRP1, Wnt5A+WIF1). The barrier function measurements were conducted in Ohms every 5 min at multiple frequencies ranging from 62.6 Hz to 64 kHz, normalized to its value at time zero, and plotted with respect to time. 40 electrodes present in each of the eight wells of 8W10E+ array traced the cells at 40 different locations in each well and the measurements were averaged.

References

1. Bernas MJ, Cardoso FL, Daley SK, Weinand ME, Campos AR, Ferreira AJG, Hoying JB, Witte MH, Brites D, Persidsky Y, et al. Establishment of primary cultures of human brain microvascular endothelial cells to provide an in vitro cellular model of the blood-brain barrier. Nat Protocols. 2010; 5:1265-1272.
2. Szulcek R, Bogaard HJ, van Nieuw Amerongen GP. Electric cell-substrate impedance sensing for the quantification of endothelial proliferation, barrier function, and motility. J Vis Exp. 2014.

3. Skaria T, Bachli E, Schoedon G. Wnt5A/Ryk signaling critically affects barrier function in human vascular endothelial cells. Cell Adh Migr. 2016:1-15.
